# Microwave-assisted ring closure reactions: Synthesis of 8-substituted xanthine derivatives and related pyrimido- and diazepinopurinediones

**DOI:** 10.1186/1860-5397-2-20

**Published:** 2006-10-27

**Authors:** Joachim C Burbiel, Jörg Hockemeyer, Christa E Müller

**Affiliations:** 1 Pharmazeutisches Institut, Rheinische Friedrich-Wilhelms-Universität Bonn, An der Immenburg 4, D-53121 Bonn, Germany

## Abstract

**Background:**

Poly-substituted xanthine derivatives are an important class of compounds in medicinal chemistry. Substitution at the 8-position of the purine ring is generally accessible by ring closure of a carboxamido-substituted uracil precursor. Although several procedures to accomplish this synthetic step have been suggested, it still remains difficult in many cases.

**Results:**

Ring closure reaction with hexamethyldisilazane was studied under microwave conditions. Reaction times were dramatically reduced by the application of microwaves in the syntheses of the 8-styrylxanthine derivative istradefylline, and in the preparation of 2-substituted pyrimido [1,2,3-*cd*]purines. Furthermore, the new procedure allowed the preparation of a previously unaccessible diazepino [1,2,3-*cd*]purine. Yields were generally improved by the new method. The addition of THF as a co-solvent proved to be crucial.

**Conclusion:**

A new, fast, and efficient ring closure method for the imidazole ring of xanthine derivatives and related tricyclic compounds has been developed. Apart from improving the syntheses of known compounds, some of which are important pharmacological tools or in development as novel drugs, it allows the preparation of 2-substituted diazepino [1,2,3-*cd*]purines – a new class of tricyclic purine derivatives.

## Background

**Figure 1:**
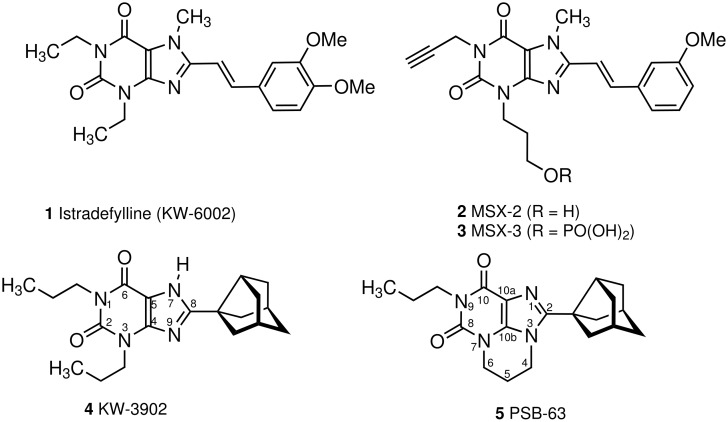
Potent, selective adenosine A_1_ and A_2A_ receptor antagonists with xanthine structure.

Xanthine derivatives are one of the most important chemical classes of adenosine receptor antagonists [1]. High affinity and selectivity for certain adenosine receptor subtypes has been achieved by introducing substituents into the 8-position of the purine ring. Styryl substitution at this position has led to A_2A_-selective antagonists, e.g. istradefylline (KW-6002, **1**) [2], MSX-2 (**2**), and its water-soluble phosphate prodrug MSX-3 (**3**) (Figure 1) [3], which are interesting drug candidates, e.g. for the therapy of Parkinsons disease [4]. In contrast, bulky cycloalkyl residues such as cyclopentyl or 3-noradamantyl (e.g. in KW-3902 (**4**), Figure 1) confer selectivity for the adenosine A_1_ receptor. A_1_-selective adenosine receptor antagonists, including **4**, are currently in clinical development for the treatment of congestive heart failure due to their positive inotropic activity combined with potent diuretic and at the same time kidney-protective properties [5]. Recently, we developed a novel synthetic access to tricyclic xanthine derivatives with pyrimido [1,2,3-*cd*]purinedione structure [6] and discovered that 2-(3-noradamantyl)-substituted derivatives, such as PSB-63 (**5**), constituted a new class of potent, selective adenosine A_1_ receptor antagonists [7]. Besides their potential use as novel drugs, the A_1_-selective antagonists KW-3902 (**4**) [8–9] and PSB-63 (**5**) [10], as well as the A_2A_-selective antagonists KW-6002 (**1**) [11–12] and MSX-2 (**2**) [13] have been widely used as pharmacological tools for studying the (patho)physiological roles and the pharmacological potential of targeting adenosine A_1_ and A_2A_ receptors, respectively.

In order to be able to perform *in vivo* studies, synthetic strategies have to be optimized and an upscaling of the procedure has to be undertaken. We have recently reported on the upscaling of the synthesis of the 8-styrylxanthine derivatives MSX-2 (**2**), MSX-3 (**3**), and KW-6002 (**1**) [14]. In the present study we have investigated whether the critical cyclization step to obtain the purine ring from a uracil precursor could be further improved by applying microwave irradiation. Furthermore, we showed that application of microwaves considerably improves the cyclization step to obtain tricyclic pyrimido [1,2,3-*cd*]purinediones. 2-Substituted diazepino [1,2,3-*cd*]purinediones, that had previously not been accessible by thermal cyclization [6] could be obtained in high yield by microwave-assisted ring closure reaction.

## Results and Discussion

**Scheme 1:**
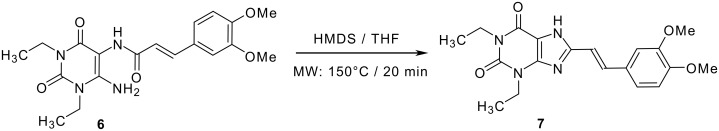
Microwave-supported synthesis of Nor-istradefylline

A key step in the classical synthetic route towards xanthines is the ring closure of the imidazole ring [15]. An improved ring closure method leading to 8-unsubstituted xanthines by microwave-assisted ring closure reaction of 5,6-diaminouracil derivatives with triethyl orthoformate was recently developed [16]. For the formation of 8-substituted xanthine derivatives, 6-amino-5-carboxamidouracil derivatives (such as compound **6**, Scheme 1) are usually condensed under acidic, alkaline or neutral conditions [3,17–18]. Although these procedures can lead to high yields of certain products, the scope of the individual method is limited and reaction conditions have to be carefully optimized for each new compound. Frequently, only low yields are obtained [19–20].

1,1,1,3,3,3-Hexamethyldisilazane (HMDS) has been shown to be a versatile condensing agent in heterocyclic ring formation [21–22]. However, its very low polarity can cause solubility problems when polar compounds are to be reacted. High temperatures (>120°C) and long reaction times (from several hours to several days) are generally required for the synthesis of xanthine derivatives, especially if uracil derivatives containing polar groups are used as precursors [3,6]. Previous experiments have shown that reaction times can be considerably shortened if HMDS is used at very high temperatures under elevated pressure (e.g. 170°C in a sealed pressure tube) [14]. The main drawback of this procedure lies in the formation of conglomerates of reagents and thus poor mixing, especially when working on a multi-gram scale.

Microwave (MW) irradiation provides the possibility of rapid heating "from within" [23], and thus can overcome the problems associated with poor mixing. Microwave-assisted synthesis has been extensively applied in the field of heterocyclic chemistry, especially when high temperatures are needed for ring formation with conventional heating [24]. Based on these findings the preparation of 8-substituted xanthine derivatives and related tricyclic derivatives was expected to be facilitated and accelerated by the application of MW heating.

### 8-Styrylxanthine derivatives

Our first target compound was istradefylline (**1**). Preliminary experiments showed that HMDS absorbs MW radiation very poorly (heating rate of 1–2°C/min at 300 W focussed MW irradiation) while in contrast the amide precursor **6** absorbs it very well (decomposition within minutes at 300 W focussed MW irradiation).

The ring closure step (**6 → 7**) in the synthesis of istradefylline had been reported to be performed by heating of **6** in a mixture of dioxane and aqueous potassium hydroxide solution; no exact reaction parameters have been described [25]. The reported yield was only about 60 % of the theoretical value since cleavage of the amide bond of **6** by the attack of hydroxide anions was a competing reaction under these conditions [18]. Initial attempts to cyclize amide **6** with HMDS under reflux conditions had failed; no reaction was observed, even after several days. Heating of the uracil-cinnamoyl-amide **6** in HMDS in the presence of a catalytic amount of ammonium sulfate at 170°C in a sealed pressure tube yielded 93 % of the xanthine derivative **7** after a long reaction time of 9 h, as previously described [14].

Our first attempts to react the poorly soluble amide **6** in HMDS using tetrahydrofuran as a co-solvent (see below) failed under conventional heating conditions, and even under drastic microwave conditions (180°C, 20 min) only a minor conversion was observed. Again, after the addition of catalytic amounts of ammonium sulfate, the reaction went smoothly and led to a high yield (92 %) of **7** (100 W, 150°C) in only 20 minutes reaction time.

**Scheme 2:**
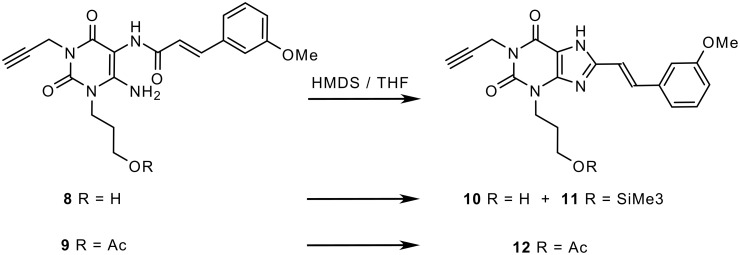
Microwave-supported synthesis of precursors of MSX-2

After this crucial improvement in the synthesis of istradefylline, we wanted to apply this method to the preparation of the related styrylxanthine derivatives MSX-2 (**2**) and MSX-3 (**3**) [3]. The ring closure reaction of **8** and **9** had previously been performed with HMDS under reflux conditions leading to xanthine derivatives **10** (MSX-1), **11** and **12**, respectively [14]. To shorten the long reaction times, the cyclization reactions of **8** and **9** were again performed under microwave irradiation as described above (Scheme 2). Unfortunately the results were less satisfying than in the former case: Although rapid formation of the xanthines **10, 11** and **12** was observed, several not identified side-products could be detected by TLC analysis, resulting in difficult isolation and purification of the desired products. This is in accordance with our previous experience that **8** and **9** tend to give multiple reaction products, and that the xanthine ring closure is seldom straightforward in these cases [3,14].

### Tricyclic xanthine derivatives

**Scheme 3:**
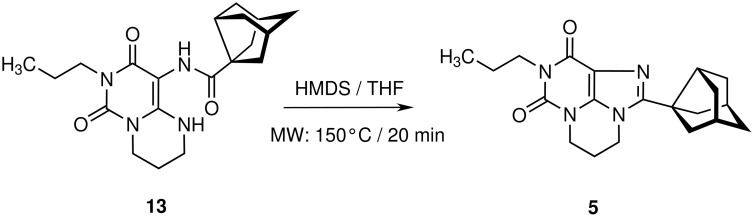
Microwave-supported synthesis of PSB-63 (**5**)

**Scheme 4:**
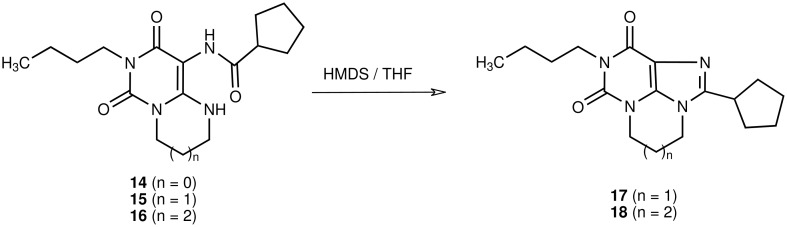
Microwave-supported synthesis of pyrimido- and diazepino-purinediones

As a next step we investigated whether the cyclization step of pyrimidopyrimidine derivatives (**13, 15**), imidazopyrimidine derivatives (**14**), and diazepinopyrimidines (**16**) to the corresponding tricyclic xanthine derivatives could be improved by the application of microwave irradiation (Scheme 3 and Scheme 4).

The cyclization of 9-(3-noradamantyl)carboxamido-6,8-dioxo-7-propyl-1,3,4,6,7,8-hexahydro-2*H*-pyrimido [1,6-*a*]pyrimidine (**13**) to the corresponding tricyclic derivative **5** was previously performed by heating it with HMDS under reflux conditions for 18 h [6]. We expected that microwave irradiation might shorten the reaction time here, too. While preliminary experiments with small amounts (80 to 200 mg) of **13** in pure HMDS were successful, the synthesis on a gram scale failed. Only poor yields of product **5** were obtained, while most of the precursor **13** remained unreacted and a small part was decomposed. Obviously the solubility of **13** in HMDS is so low that the reaction can only take place at the solid-liquid interface. This hypothesis is supported by our finding that heating of amide **13** in HMDS at elevated temperatures (120–140°C) in a glass pressure tube led to the formation of two separated layers of liquids and no conversion at all, even after several days. To achieve higher homogeneity, a solution of **13** in tetrahydrofuran (THF) was prepared. A clay-like composition evolved in the microwave vial when HMDS was added under careful stirring. The resulting fine suspension absorbed MW radiation very well. After 20 min at 100 W product **5** could be isolated in a yield of 75%, even on a gram scale. This procedure led to a high internal pressure in the microwave vial (10 bar at 140°C) due to the low boiling point of THF. In contrast to the ring closure reaction yielding 8-styrylxanthine derivative **7**, in the case of pyrimidopurine derivative **5** the addition of ammonium sulfate did not affect the reaction at all.

Subsequently, we further investigated the scope of the method. We had previously demonstrated that pyrimido [1,6-*a*]pyrimidine derivatives like **13** and **15** could be converted to the tricyclic pyrimido [1,2,3-*cd*]purine derivatives by refluxing in HMDS, while the analogous ring closure reactions of imidazo [1,2-*c*]pyrimidines (e.g. **14**) to the corresponding imidazo [1,2,3-*cd*]purine derivatives, and of pyrimido [1,6-a][1,3]diazepines (e.g. **16**) to the diazepino [1,2,3-*cd*]purine derivatives failed under these conditions (Table 1) [6]. When conducting these reactions under microwave conditions, we found that indeed harsher conditions were required for the ring closure of the 5-6-7-ring system **18** (200 W, 160°C, 20 min) than for the 5-6-6-ring system **17** (100 W, 140°C, 20 min). Nevertheless, the 7-ring derivative could be obtained in very high yield (91 %) (Scheme 4/Table 1). In contrast, no ring closure products could be detected for the 5-5-6 ring system, even at 160°C (300 W), regardless of the addition of ammonium sulfate. Some compounds of this kind have been prepared by closing the aliphatic ring as a final step by *Simo* et al. [26]

**Table 1:** Synthesis of tricyclic xanthine derivatives

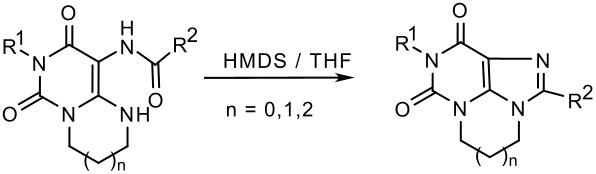						
Compounds					Yield [%]	
Reactand	Product	R^1^	R^2^	Size^A^	Reflux^B^	Microwave^C^

13	5	Pr	nor-adamantyl	6	60	75
14	-	Bu	cyclopentyl	5	0	0
15	17	Bu	cyclopentyl	6	60	98
16	18	Bu	cyclopentyl	7	0	91

A: size of non-aromatic ring. B: 18 h, yields taken from literature [6]. C: see text for exact conditions.

In fact, this is the first access to 2-substituted diazepino [1,2,3-*cd*]purines. 2-Unsubstituted derivatives have previously been described by reaction of 10-amino-2,3,4,5-tetrahydropyrimido [1,6-*a*][1,3]diazepine-7,9-dione with triethyl orthoformate, but the reported synthetic procedure does not allow the preparation of 2-substituted derivatives [27].

In conclusion, we have significantly improved HMDS-mediated ring closure reactions for the preparation of adenosine receptor antagonists, namely, the 8-styrylxanthine derivative istradefylline (**1**), an A_2A_-selective antagonist currently in clinical development as a novel treatment for Parkinson's disease, and 2-cycloalkyl-substituted pyrimido [1,2,3-*cd*]purines, such as PSB-63 (**5**), an A_1_-selective antagonist that is used as a pharmacological tool. Reaction times were dramatically shortened by applying microwave irradiation to a solution or suspension of the starting compounds in a mixture of HMDS and tetrahydrofuran. The addition of THF as a co-solvent proved to be crucial. Yields were generally improved by the new method. The new microwave-assisted cyclization procedure allowed the preparation of a previously unaccessible 2-substituted diazepino [1,2,3-*cd*]purine (**18**), and may therefore open a general access to the new class of 2-substituted diazepino [1,2,3-*cd*]purines.

## Experimental

### General Procedures

All microwave reactions were carried out in 10 ml sealed glass tubes in a focused mono-mode microwave oven ("Discover" by CEM Corporation, Matthews, NC). Maximum power levels, target temperatures and reaction times are given. All commercially available reagents and solvents were used without further purification. NMR spectra were determined on a Bruker Avance 500 MHz spectrometer.

### 8- [2(E)-(3,4-Dimethoxyphenyl)vinyl]-1,3-diethylxanthine (7) [2]

To a suspension of 1.17 g (3.0 mmol) of 6-amino-5-(3,4-dimethoxycinnamoylamino)-1,3-diethyluracil (**6**) [14] in THF (3 ml) in a 10 ml pressure vial 0.1 g of finely ground (NH_4_)_2_SO_4_ and HMDS (2 ml, 10 mmol) were added, forming a viscous yellow suspension. After microwave irradiation (100 W, 150°C, 20 min) the reaction mixture was hydrolyzed by the addition of excess methanol (ca. 10 ml), concentrated under vacuo and treated with ethyl acetate (40 mL). The colorless precipitate was filtered off under reduced pressure, washed with ethyl acetate (40 ml) and dried at 70°C (92% yield, purity as determined by NMR: >98% (for spectral data see [14])). (^1^H-NMR [see Supporting Information File 1] and ^13^C-NMR [see Supporting Information File 2] are added as additional files)

### 2-(3-Noradamantyl)-4,5-dihydro-9-propyl-6*H*,8*H*-pyrimido [1,2,3-*cd*]purine-8,10(9*H*)-dione (5) [6]

To a suspension of 9-(3-noradamantyl)carboxamido-6,8-dioxo-7-propyl-1,3,4,6,7,8-hexahydro-2H-pyrimido [1,6-*a*]pyrimidine (**13**) [6] (1.0 g, 8.4 mmol) in THF (3 ml) in a 10 ml pressure vial, HMDS (2 ml) was added. Microwave irradiation was applied (100 W, 140°C) for 20 min. The resulting yellow solution was hydrolyzed with 6 ml of methanol while still warm (ca. 50°C). After the formation of ammonia gas had ceased, the product was filtered off under reduced pressure, washed with ethyl acetate (20 ml) and subsequently with diethyl ether (10 ml). Compound **1** (0.74 g, 75%) was obtained as an off-white solid (purity as determined by NMR: >97%). (^1^H-NMR [see Supporting Information File 3] and ^13^C-NMR [see Supporting Information File 4] are added as additional files)

### 9-Butyl-2-cyclopentyl-4,5-dihydro-6*H*,8*H*-pyrimido [1,2,3-*cd*]purine-8,10(9*H*)-dione (17) [6]

To a solution of 7-butyl-9-cyclopentanecarboxamido-6,8-dioxo-1,3,4,6,7,8-hexahydro-2*H*-pyrimido [1,6-*a*]pyrimidine (**15**) [6] (0.17 g, 0.5 mmol) in THF (1 ml) in a 10 ml pressure vial, HMDS (1 ml) was added. Microwave irradiation was applied (100 W, 140°C) for 20 min. The resulting yellow solution was hydrolyzed with 4 ml of methanol while still warm (ca. 50°C). After the formation of ammonia gas had ceased, the liquid phase was distilled off under reduced pressure. Compound **17** (0.16 g, 98%) was obtained as an off-white solid (purity as determined by NMR: >95%). (^1^H-NMR [see Supporting Information File 5] and ^13^C-NMR [see Supporting Information File 6] are added as additional files)

### 3-Butyl-11-cyclopentyl-6,7,8,9-tetrahydrodiazepino [1,2,3-*cd*]purine-2,4-dione (18)

To a solution of 8-butyl-10-cyclopentanecarboxamido-1,2,3,4,5,7,8,9-octahydropyrimido [1,6-c][1,3]diazepine-7,9-dione (**16**) [6] (0.17 g, 0.5 mmol) in THF (1 ml) in a 10 ml pressure vial, HMDS (1 ml) was added. Microwave irradiation was applied (200 W, 160°C) for 20 min. The resulting yellow solution was hydrolyzed with 4 ml of methanol while still warm (ca. 50°C). After the formation of ammonia gas had ceased, the liquid phase was distilled off under reduced pressure. The product was purified over a short silica gel column (5% EtOH in CH_2_Cl_2_ as eluent). Compound **18** (0.15 g, 91%) was obtained as a yellow oil (purity as determined by NMR: >95%). ^1^H-NMR (500 MHz, CDCl_3_) δ = 0.90 (t, 3H, *J* = 7.4 Hz, CH_3_), 1.35 (sex, *J* = 7.4 Hz, 2H), 1.61 (m, 4H), 1.81 (m, 2H), 1.99 (m, 4H), 2.16 (quint, 4H, *J* = 2.8 Hz), 3.04 (quint, 1H, *J* = 8.1 Hz), 3.99 (dd, 2H, *J* = 7.3 Hz, *J* = 7.4 Hz), 4.16 (m, 2H), 4.31 (m, 2H). ^13^C-NMR (500 MHz, CDCl_3_) δ = 13.83, 20.14, 25.23, 25.54, 25.61, 30.07, 31.37, 37.12, 41.57, 42.82, 43.46, 115.57, 141.03, 151.17, 151.66, 157.24. (^1^H-NMR [see Supporting Information File 7], ^13^C-NMR [see Supporting Information File 8], and IR [see Supporting Information File 9] spectra are added as additional files)

## Supporting Information

File 1 ^1^H-NMR of compound 5

File 2 ^13^C-NMR of compound 5

File 3 ^1^H-NMR of compound 5

File 4 ^13^C-NMR of compound 5

File 5 ^1^H-NMR of compound 17

File 6 ^13^C-NMR of compound 17

File 7 ^1^H-NMR of compound 18

File 8 ^13^C-NMR of compound 18

File 9 IR-spectrum of compound 18
