# Supplementary figures and images for: Microwave-assisted ring closure reactions: Synthesis of 8-substituted xanthine derivatives and related pyrimido- and diazepinopurinediones
Source: Beilstein J Org Chem. 2006 Oct 27;2:20. doi: 10.1186/1860-5397-2-20 (PMC1698928; doi:10.1186/1860-5397-2-20)

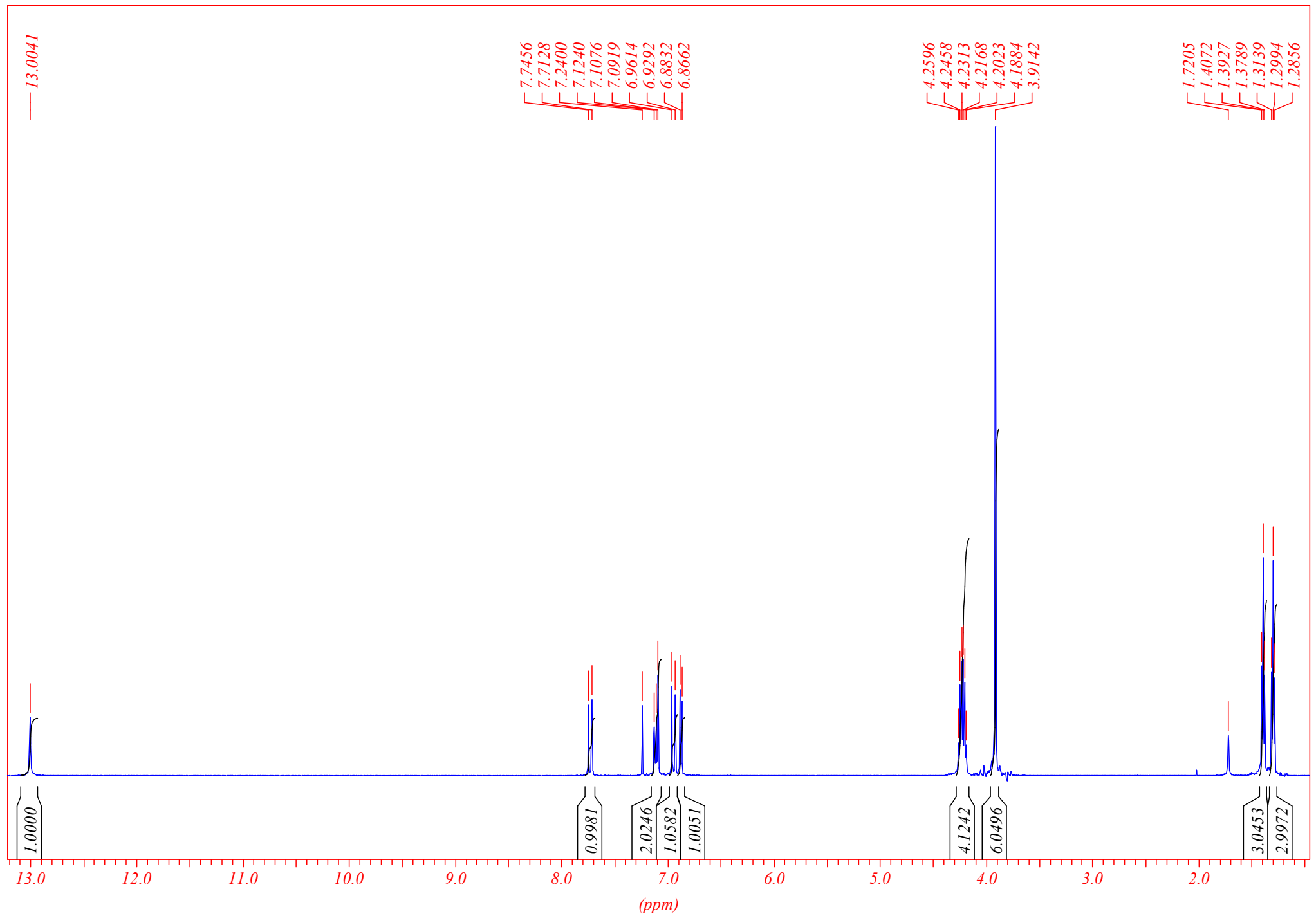

\*\*\* Cur  
NAME  
EXPNO  
PROCNO

Supplement: File 1 — 1H-NMR of compound 5 [file Beilstein_J_Org_Chem-02-20-s001.pdf]

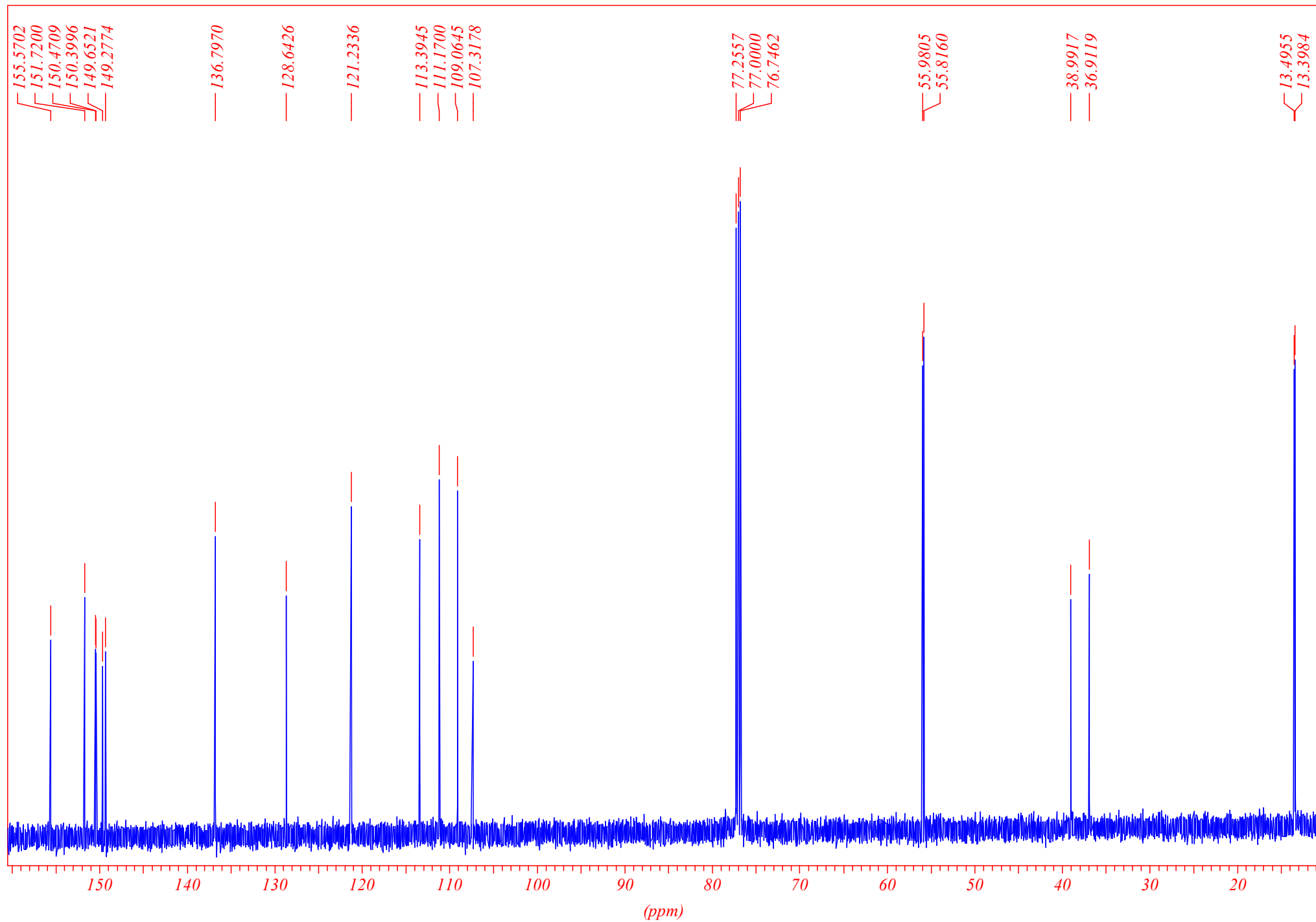

\*\*\* Curr  
NAME  
EXPNO  
PROCNO

Supplement: File 2 — 13C-NMR of compound 5 [file Beilstein_J_Org_Chem-02-20-s002.pdf]

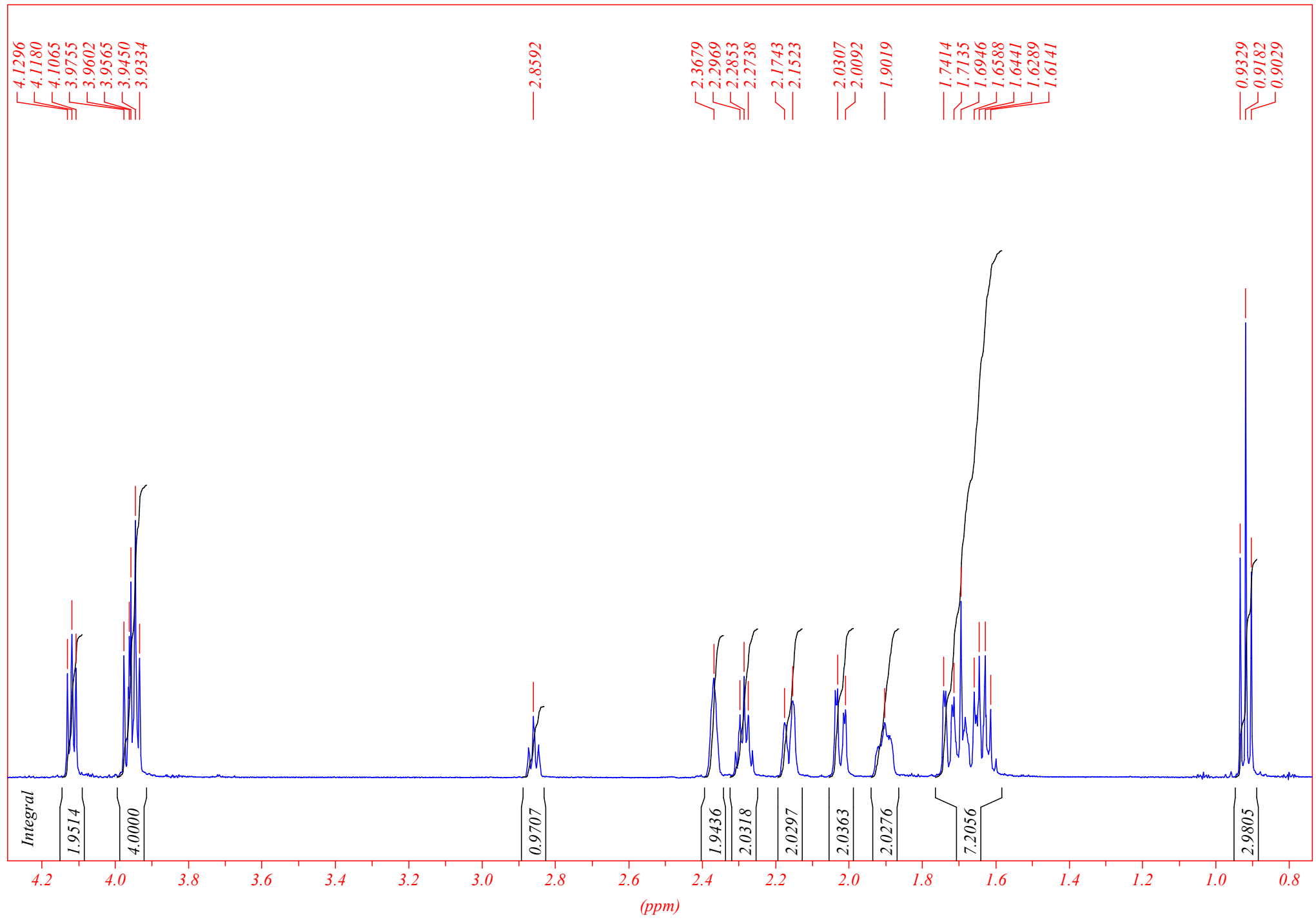

\*\*\* Curr  
NAME  
EXPNO  
PROCNO

Supplement: File 3 — 1H-NMR of compound 5 [file Beilstein_J_Org_Chem-02-20-s003.pdf]

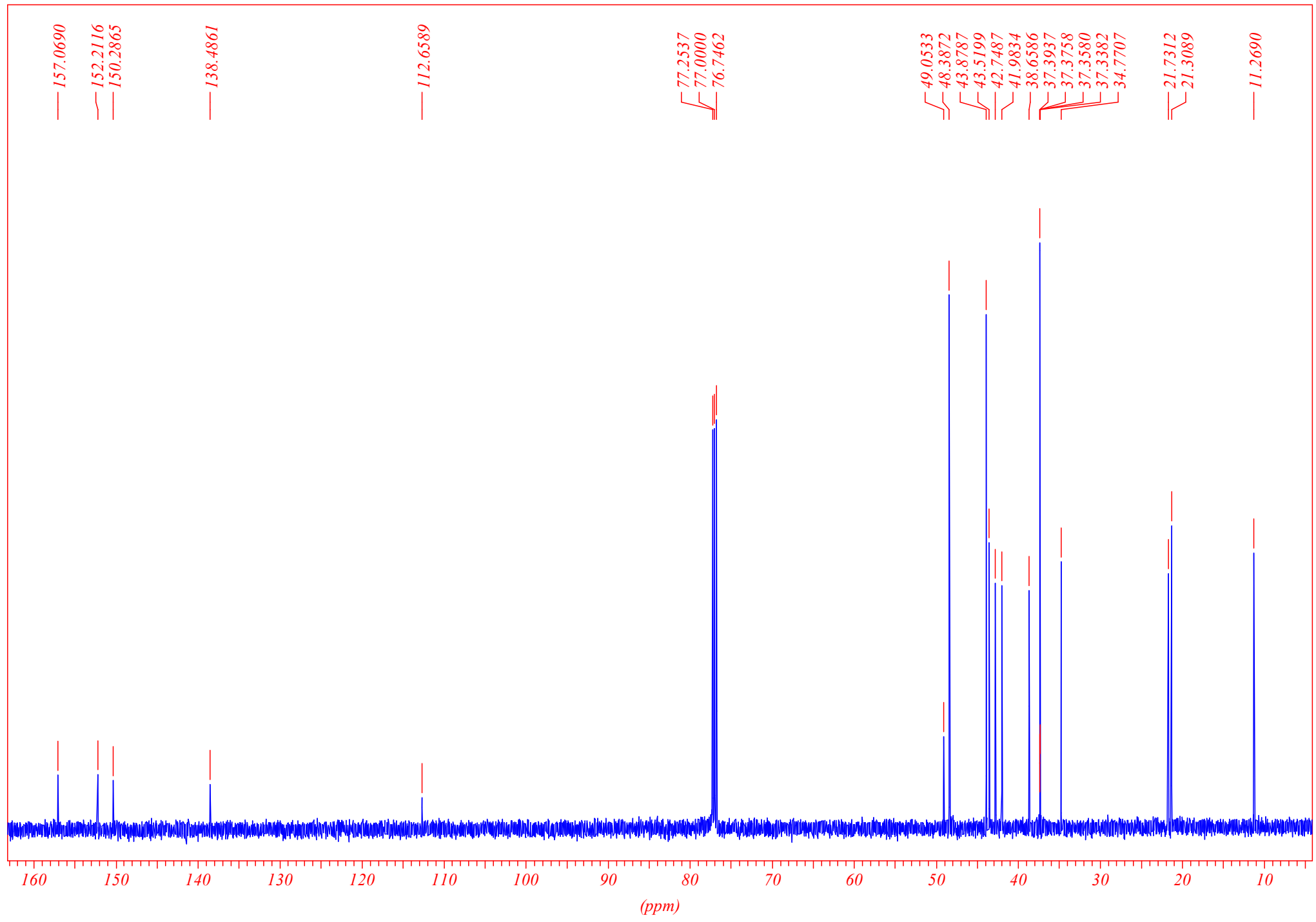

\*\*\* Curr  
NAME  
EXPNO  
PROCNO

Supplement: File 4 — 13C-NMR of compound 5 [file Beilstein_J_Org_Chem-02-20-s004.pdf]

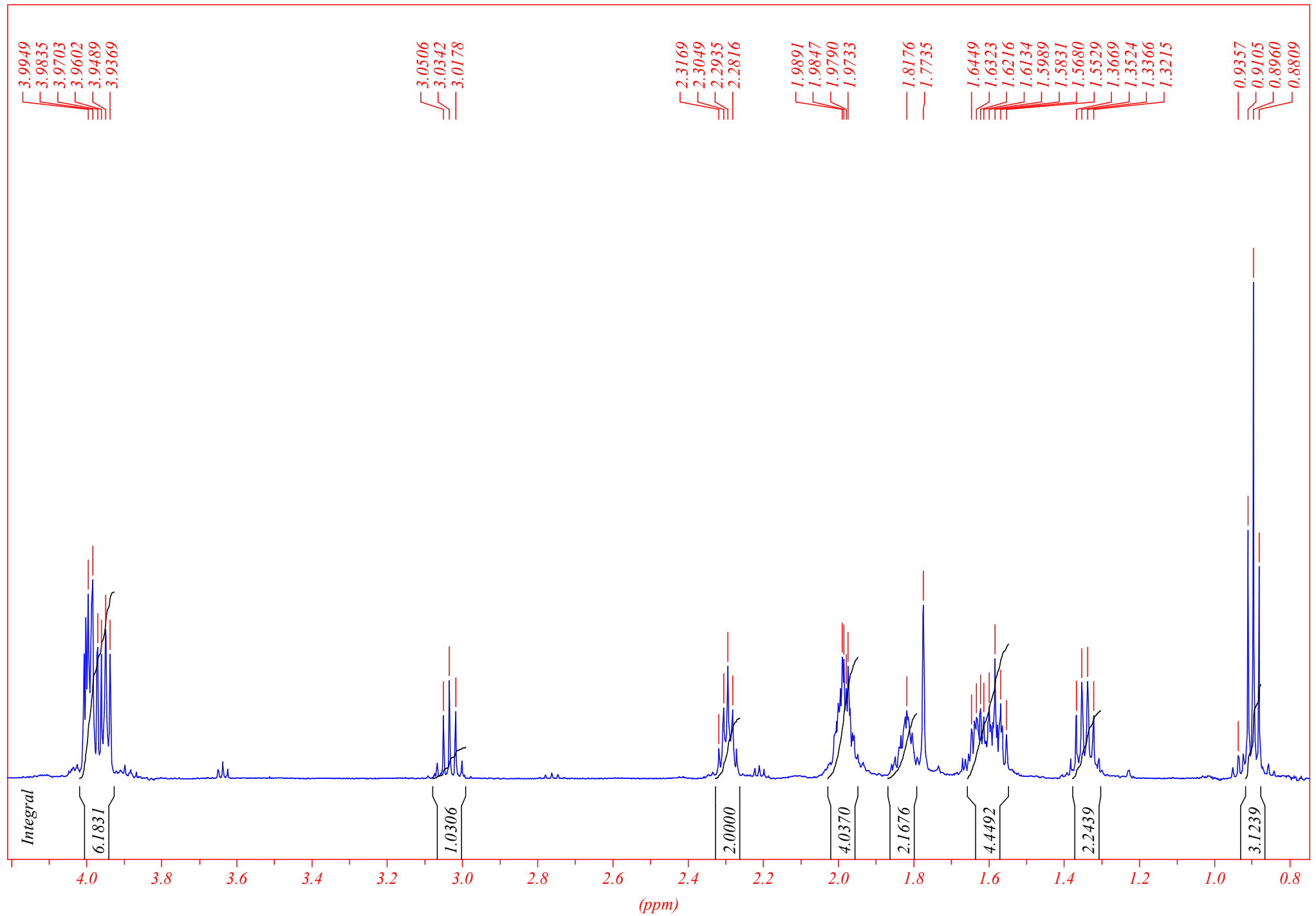

\*\*\* Curr  
NAME  
EXPNO  
PROCNO

Supplement: File 5 — 1H-NMR of compound 17 [file Beilstein_J_Org_Chem-02-20-s005.pdf]

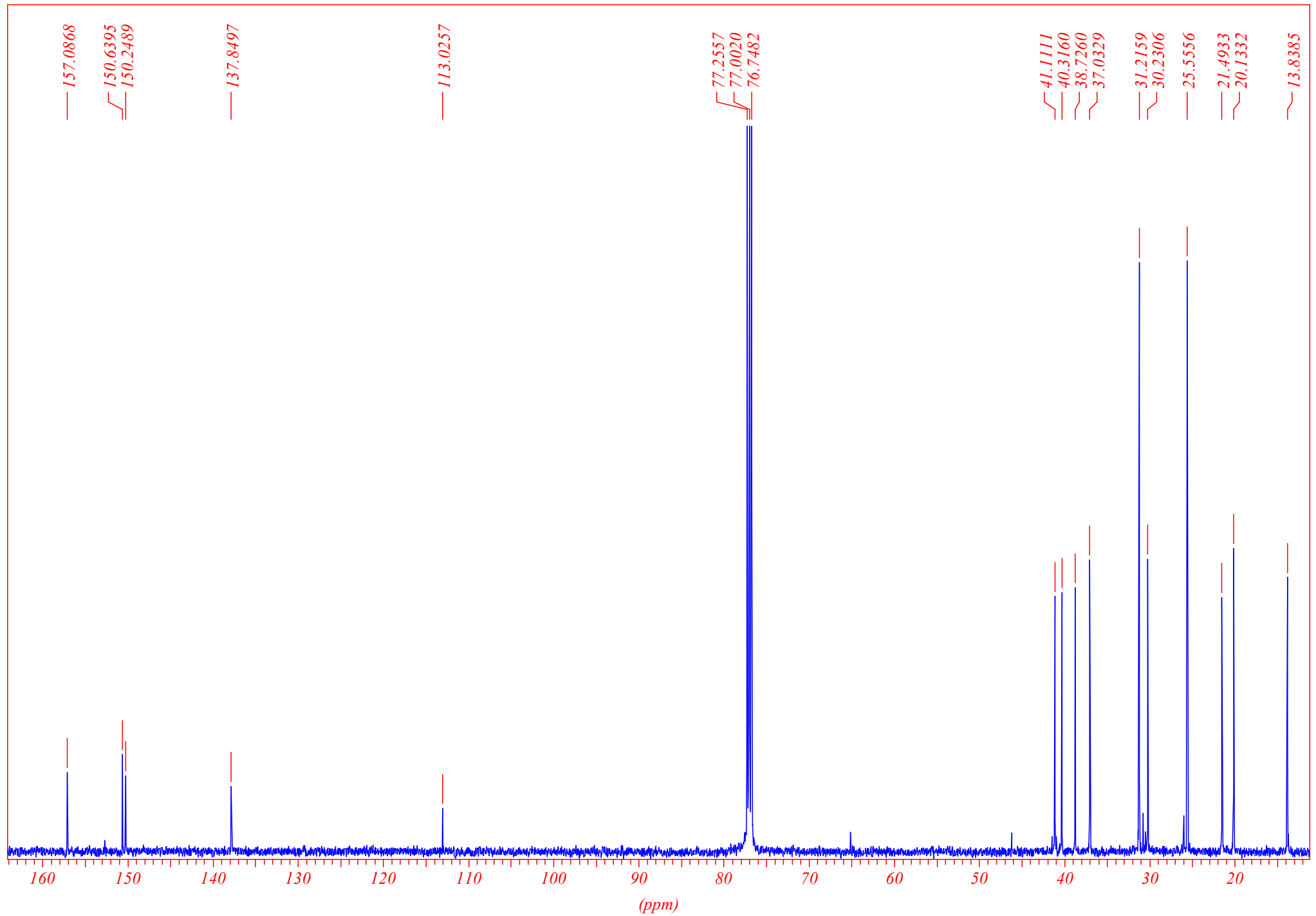

\*\*\* Curr  
NAME  
EXPNO  
PROCNO

Supplement: File 6 — 13C-NMR of compound 17 [file Beilstein_J_Org_Chem-02-20-s006.pdf]

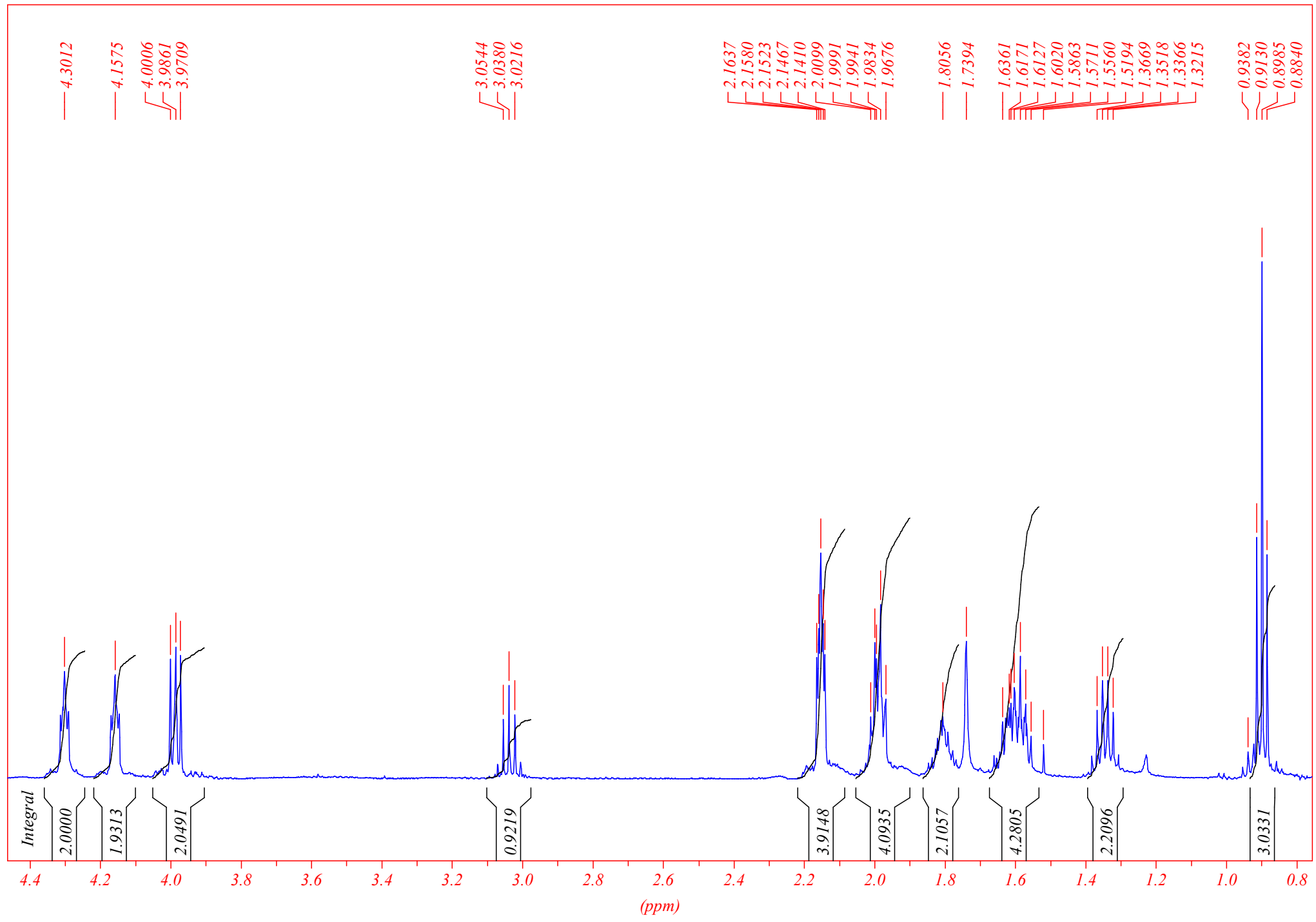

\*\*\* Curr  
NAME  
EXPNO  
PROCNO

Supplement: File 7 — 1H-NMR of compound 18 [file Beilstein_J_Org_Chem-02-20-s007.pdf]

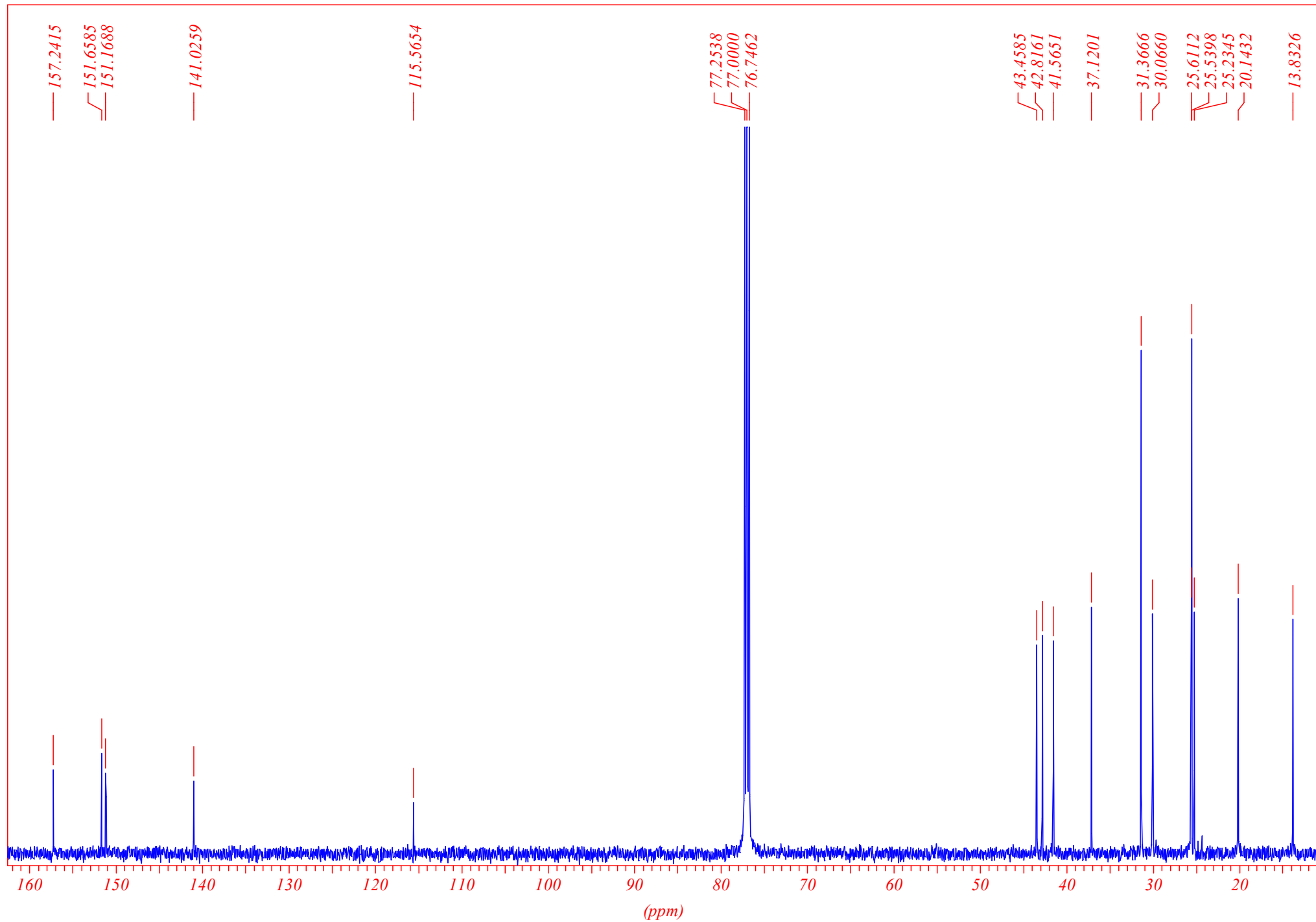

\*\*\* Curr  
NAME  
EXPNO  
PROCNO

Supplement: File 8 — 13C-NMR of compound 18 [file Beilstein_J_Org_Chem-02-20-s008.pdf]

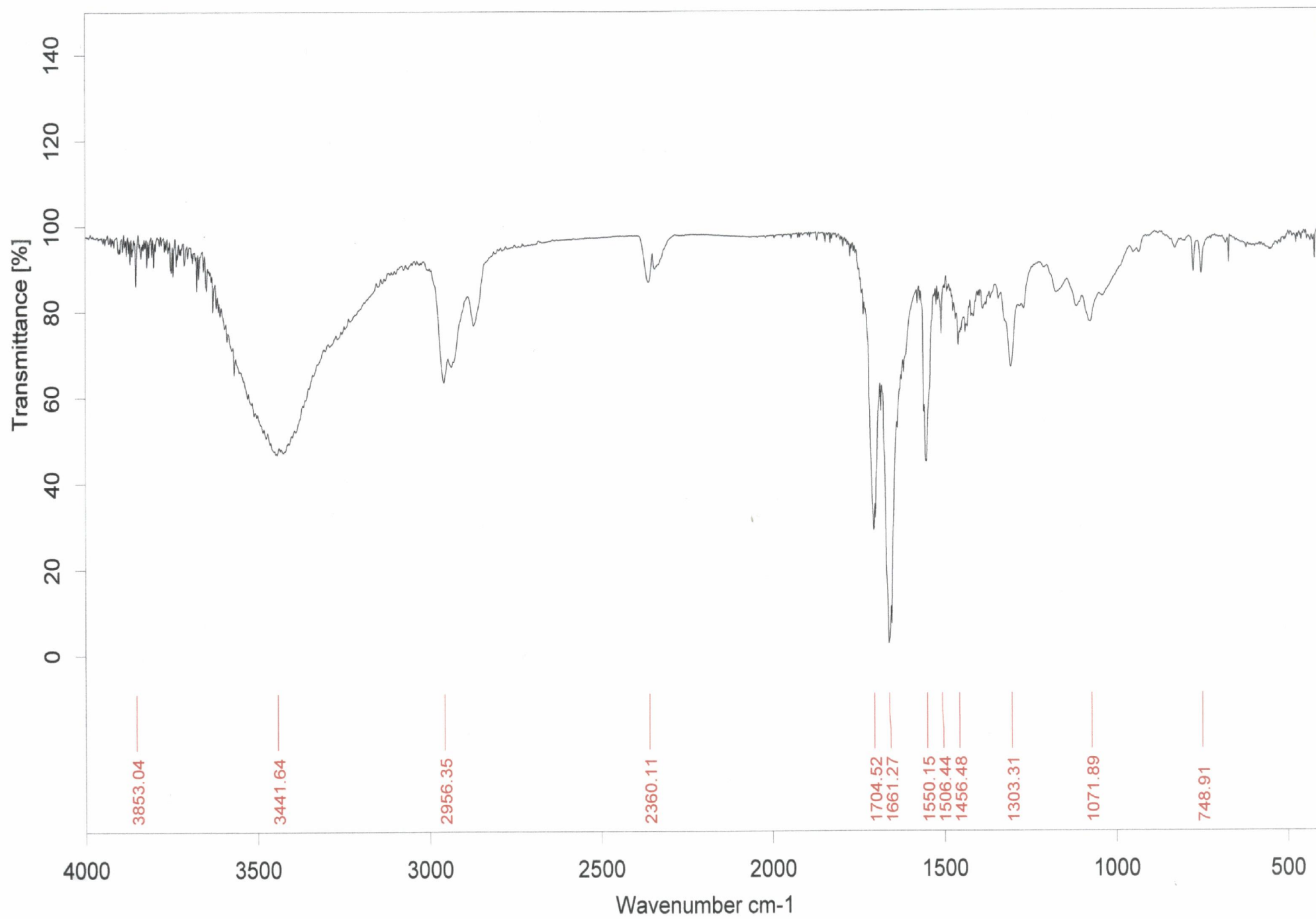

Supplement: File 9 — IR-spectrum of compound 18 [file Beilstein_J_Org_Chem-02-20-s009.pdf]
